# Capsid Mutated Adeno-Associated Virus Delivered to the Anterior Chamber Results in Efficient Transduction of Trabecular Meshwork in Mouse and Rat

**DOI:** 10.1371/journal.pone.0128759

**Published:** 2015-06-08

**Authors:** Barbara Bogner, Sanford L. Boye, Seok Hong Min, James J. Peterson, Qing Ruan, Zhonghong Zhang, Herbert A. Reitsamer, William W. Hauswirth, Shannon E. Boye

**Affiliations:** 1 Department of Ophthalmology and Optometry, SALK/Paracelsus Medical University, Salzburg, Austria; 2 Department of Ophthalmology, University of Florida, Gainesville, United States of America; University Pierre, FRANCE

## Abstract

**Background:**

Adeno associated virus (AAV) is well known for its ability to deliver transgenes to retina and to mediate improvements in animal models and patients with inherited retinal disease. Although the field is less advanced, there is growing interest in AAV’s ability to target cells of the anterior segment. The purpose of our study was to fully articulate a reliable and reproducible method for injecting the anterior chamber (AC) of mice and rats and to investigate the transduction profiles of AAV2- and AAV8-based capsid mutants containing self-complementary (sc) genomes in the anterior segment of the eye.

**Methodology/Principle Findings:**

AC injections were performed in C57BL/6 mice and Sprague Dawley rats. The cornea was punctured anterior of the iridocorneal angle. To seal the puncture site and to prevent reflux an air bubble was created in the AC. scAAVs expressing GFP were injected and transduction was evaluated by immunohistochemistry. Both parent serotype and capsid modifications affected expression. scAAV2- based vectors mediated efficient GFP-signal in the corneal endothelium, ciliary non-pigmented epithelium (NPE), iris and chamber angle including trabecular meshwork, with scAAV2(Y444F) and scAAV2(triple) being the most efficient.

**Conclusions/Significance:**

This is the first study to semi quantitatively evaluate transduction of anterior segment tissues following injection of capsid-mutated AAV vectors. scAAV2- based vectors transduced corneal endothelium, ciliary NPE, iris and trabecular meshwork more effectively than scAAV8-based vectors. Mutagenesis of surface-exposed tyrosine residues greatly enhanced transduction efficiency of scAAV2 in these tissues. The number of Y-F mutations was not directly proportional to transduction efficiency, however, suggesting that proteosomal avoidance alone may not be sufficient. These results are applicable to the development of targeted, gene-based strategies to investigate pathological processes of the anterior segment and may be applied toward the development of gene-based therapies for glaucoma and acquired or inherited corneal anomalies.

## Introduction

Adeno associated virus (AAV)- mediated gene delivery has been used successfully to improve vision in animal models of inherited retinal disease and its safety/efficacy has also been proven in clinical trials [1–8]. In addition, AAV has been used to create animal models and investigate pathological mechanisms of ocular diseases e.g. in optic neuropathy [9] or age-related macular degeneration [10]. While transduction of the outer and inner retina is achievable via subretinal and intravitreal injection of AAV, respectively [11–13], these injection routes are not capable of, or at best, ill-suited for transducing tissues of the anterior segment. While the field is less advanced, there is a growing interest in targeting tissues like the trabecular meshwork (TM), which plays a role in the pathophysiology of glaucoma (reviewed in [14–17], and corneal layers, which can be affected by genetically determined non-inflammatory corneal dystrophies (reviewed in [18, 19]). Among others, targets of interest within the TM include pro-fribrotic and microfibril associated genes such transforming growth factor- beta (*TGFB*), connective tissue growth factor (*CTGF*), fibronectin (*FN*), bone morphogenic protein (*BMP*) and tissue transglutaminase (*tTG*) [15, 20–31]. Targets of interest in the cornea include carbohydrate sulfotransferase 6 (*CHST6*), keratin 3 (*KRT3*), keratin 12 (*KRT12*), a FYVE finger containing phosphoinositide kinase (*PIK5K3*), sodium bicarbonate transporter-like protein (*SLC4A11*), tumor associated calcium signal transducer 2 (*TACSTD2*), transforming growth factor, beta induced, 68 kDA (*TGFBI*) and UbiA prenyltransferase domain-containing protein 1 (*UBIAD1*) [18, 19]. Gene delivery to these tissues will require both a reliable injection technique and vectors that can efficiently transduce target cells *in vivo*. While other vectors have successfully been used to transduce these tissues [31–49], AAV is the preferred platform due to its inherent lack of immunogenicity, persistent transgene expression, ease of use (very rapid and facile cloning of vector constructs relative to other vector platforms) and clinical relevance in the eye.

Different AC (intracameral) injection methods have been described for delivering AAV in cynomolgus macaques, rats and mice [50–52]. Due to their small size and shallow anterior chambers, AC injections in mouse eyes are more challenging [52]. Gene delivery to outflow pathways via AC injection is preferred to intravitreal injection given the relatively small volume of the former. AC injection allows direct access to target tissues like TM and cornea with relatively little dilution effect (mouse AC volume is ~3 μl, mouse vitreous volume is ~10 μl) [53]. To date, all reports of AAV-mediated transduction of TM or cornea have focused on unmodified, first generation serotypes AAV1-AAV9 [49, 51, 54–58]. With the goal of targeting transgene expression to these tissues, we chose to focus on two ‘parent’ capsid serotypes, AAV2 and AAV8, as each has previously demonstrated utility for targeting TM and cornea, respectively [49, 51, 54, 57]. AAV2 transduces TM cells *in vitro*, organoculture [54], and TM in rat and non-human primate after intracameral injection [51]. The primary receptor for AAV2 is heparan sulfate proteoglycan (HSPG) [59, 60]. An abundance of HSPGs are found in the extracellular matrix and basement membranes of the TM, as is AAV2’s co-receptor αVβ5 integrin [61]. HSPGs are also present in cornea [62]. Conversely, AAV8 does not bind HSPG [63]. However, studies show that this serotype efficiently transduces corneal stroma [27,29]. Thus, our selection was based on known AAV receptor biology and glycan footprints in target tissues.

The purpose of the current study was twofold. First, we aimed to investigate whether capsid mutated AAV vectors delivered via AC injection could efficiently transduce tissues of the anterior segment including TM and cornea. Our standard AAV vector vehicle (BSS supplemented with Tween 20) is low viscosity. We purposefully avoided using a high viscosity vehicle for injection because such ‘gels’ are composed of glycosaminoglycans (GAGs), which are the very substrates that AAV capsid’s bind. Due to the low viscosity of injection solution and the small size/shallow anterior chamber in rodents, the propensity for vector reflux during injection was high. Our second aim, therefore, was to describe an anterior chamber injection method that was well tolerated and gave generally reproducible results in mice and rats with the goal of providing sufficient methodological detail for any labs interested in attempting similar experiments.

## Methods

### Experimental Animals

To establish the injection procedure and to investigate transduction profiles, male and female C57BL/6 mice and Sprague Dawley rats were used. All animal procedures were approved by the Institutional Animal Care and Use Committee on the Ethics of Animal Experiments of the University of Florida, Gainesville (IACUC Protocol # 201101103) and were carried out in strict accordance with the recommendations of the Association for Research in Vision and Ophthalmology (ARVO) Statement for the Use of Animals in Ophthalmic and Vision Research and the Guidelines for the Care and Use of Laboratory Animals of the National Institutes of Health. To ameliorate any pain or distress during ocular injections, rodents were anesthetized with intraperitoneal injection of ketamine/xylazine at a mixture of ketamine 80–120 mg/kg, xylazine 10–15 mg/kg. Rodents were humanely sacrificed by sedation first with ketamine/xylazine (as outlined above) followed by cervical dislocation in accordance with the University of Florida IACUC guidelines.

### AAV-Vector Construction and Production

All scAAV serotypes and mutants contain the ubiquitous, truncated chimeric CMV-chicken β-actin (smCBA) promoter [64] driving the green fluorescent protein (GFP) reporter cDNA. AAV2 and AAV8 capsid mutants were generated by directed mutagenesis of highly conserved surface-exposed tyrosine and threonine residues with the QuickChange Multi Site-Directed Mutagenesis Kit (Agilent Technologies, CA 200514) as previously described[65–67]. Selected tyrosine and threonine residues were mutated to phenylalanine (Y−F) or valine (T−V), respectively. A summary of mutated residues and vector nomenclature are described in Table 1.

**Table 1:** AAV vectors and titers.

Construct	AAV Serotype	Titer (vg/mL)
sc-smCBA-hGFP	AAV2	~2x10^12^
sc-smCBA-hGFP	AAV2(Y444F)	~9x10^11^
sc-smCBA-hGFP	AAV2(Y444+500+730) (aka "AAV2(triple)")	~3x10^12^
sc-smCBA-hGFP	AAV2(Y252+272+444+500+700+704+730F) (a.k.a. "AAV2(septuple)")	~2x10^12^
sc-smCBA-hGFP	AAV8(Y733F)	~2x10^12^

scAAV vector preparations were performed by the 3-plasmid, co-transfection method according to methods described in detail previously [68–70]. Briefly, a calcium phosphate precipitation transfection was set up by mixing 500 μg: of vector plasmid “sc-trs-SB-smCBA-hGFP”, 650 μg of plasmid coding for AAV2 ‘rep’ and the variant specific ‘cap’ and 1550 μg of helper plasmid “pXX6” containing Adenovirus helper genes, respectively (**Table 1**). scAAV vector was titered for DNase-resistant vector genomes by quantitative real-time PCR against a known standard. Resulting titers are contained in Table 1.

### Anterior Chamber Injections. Anterior Chamber Injections

**Fig 1:**
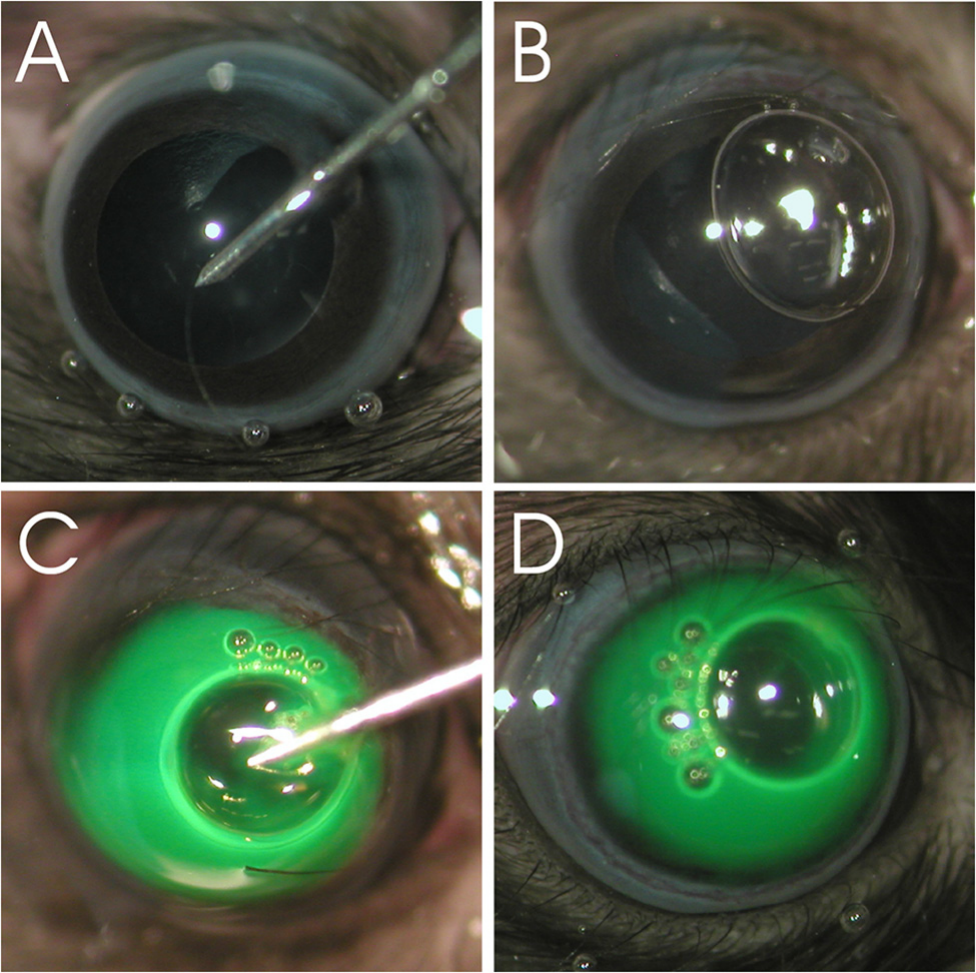
Injection procedure in mice. (A) The cornea is punctured closely anterior to the iridocorneal angle. (B) Before vector administration, an air bubble is created to seal the puncture site. (C-D) scAAV or vehicle dyed with fluorescein is injected. The air bubble seals the puncture site and reflux is minimized. The air bubble is absorbed within 24 hrs.

AC injections were performed in C57BL/6 mice (injection volume 1 μl; age ~5 weeks; The Jackson Laboratory, Maine, USA) and Sprague Dawley rats (injection volume 2 μl; age ~12 weeks, Harlan Laboratories, USA). Mice (n = 35) and rats (n = 35) were anesthetized with a mixture of 100 mg/kg ketamine, 20 mg/kg xylazine and saline. Pupils were dilated with 1% atropine sulfate (Akorn, IL, USA) and 2.5% phenylephrine hydrochloride (Akorn, IL, USA). One drop of 0.5% proparacaine hydrochloride (Alcon, TX, USA) was applied to the cornea as a topical anesthetic. Animals were positioned slightly lateral and placed under a surgical microscope (Nikon SMZ800 fitted with Olympus Z4040Zoom camera). Hypromellose ophthalmic demulcent solution 2.5% (Gonak; Acorn, IL, USA) was applied onto the cornea to improve visibility when manipulating in the AC. Cornea was punctured with a 33-gauge needle (beveled 25° angle, 3-sharpening, with bevel up) just anterior of the iridocorneal angle. Care was taken not to disturb the iris or the lens (**Fig 1**). With a beveled 33-gauge needle we slowly injected approximately 200 nl air through the same puncture site at a rate of 30 nl/s. The needle was kept in position until the air bubble was fully formed and then slowly removed to facilitate gentle movement of the air bubble towards the puncture site (**Fig 1B**). When the bevel of the needle was close to the puncture site, it was pulled out quickly to keep the bubble at the puncture site. Then, scAAV vectors expressing GFP under the constitutive small CMV-chicken β-actin (smCBA) promoter or vehicle control (BSS with 0.014% Tween-20) were injected via the same puncture site with the intraocular injection kit (WPI, Sarasota, USA) connected to a beveled, 33 gauge needle using a precision micropump (WPI, Sarasota, USA) (**Fig 1C, Table 2**). Fluorescein was added to each injection solution to visualize injections. To help the virus to diffuse away from the needle, it was infused slowly (30 nl/s in mice, 45 nl/s in rats). Before the needle was removed, it was held in position for 1 min. and care was taken to keep the air bubble at the puncture site in order to prevent reflux of vector/vehicle control. In some cases, repositioning of the air bubble was necessary to ensure a good seal. Neomycin, polymyxin B sulfate and dexamethasone ointment (Alcon, TX, USA) were applied to the eye after the procedure. Animals were placed on absorbent paper atop a 37°C heating plate for recovery and were then returned to their cages.

**Table 2:** Experimental groups of titer-matched scAAV-injected and mock-injected eyes.

Group	AAV Serotype	n
1	AAV2	5
2	AAV2(Y444F)	5
3	AAV2(triple)	5
4	AAV2(septuple)	5
5	AAV8(Y733F)	5
6	vehicle control	5
7	no injection	5

A sample size of 5 eyes per group was analyzed by immunohistochemistry for GFP-expression. The experimental protocol was performed in C57BL/6 mice and Sprague Dawley rats.

### Immunohistochemistry

Eyes from scAAV-treated or mock-injected mice/rats were enucleated 4 weeks post-injection and fixed in 4% paraformaldehyde (PFA) for 1 to 2 hrs. Before the eyes were rinsed in 1xPBS (overnight) and soaked in 30% sucrose in 1xPBS (at least 12 hrs.) they were separated slightly anterior of the equator into the anterior and the posterior segment and the lens was carefully removed. Then, the divided eyes were immersed in OCT (Tissue Tek OCT 4583: Sakura Finetek USA, Inc., Torrance, CA), quick frozen in a bath of dry ice/ethanol and stored at -20°C until sectioning. Per eye, eight 10 micron cross sections in different planes were obtained by moving through tissue in a lateral to medial direction. For immunohistochemistry, all samples were prepared in parallel and processed identically. The sections from the anterior part of the eye were permeated and blocked with TBS containing 1% BSA, 0.1% Triton-X100 and species-matched normal serum for 1 hr. at room temperature (RT). To enhance the endogenous GFP-expression signal, sections were incubated with rabbit polyclonal GFP antibody [71] (1:1000, generously provided by Dr. Clay Smith, University of Florida) over night at 4°C. Alexa 488-conjugated secondary antibody (1:1000; Life Technologies, Germany) was applied for 1 hr. at RT. Nuclei were counterstained with 4',6-diamidino-2-phenylindole (DAPI) (1:2000; Merck, Austria). Between each incubation step and after DAPI application, washing steps of 3 x 5 min. were performed. The slides were mounted in TBS-Glycerin pH 8.6 and analyzed by confocal microsopy (LSM 710, Zeiss, Germany). In a subset of animals GFP and Thrombospondin-1 (TSP-1) double-labeling was performed to identify TM structures. TSP-1 is a matricellular protein (= extracellular protein modulating cell function and regulating cell surface and matrix interaction [72, 73]) that is produced and secreted by TM cells and is described to be present in the juxtacanalicular TM region [20, 74] and throughout the TM [75]. It was thus used as a marker to distinguish TM structures from others in the anterior chamber angle. Sections were incubated with mouse monoclonal TSP-1 antibody (clone A6.1 1:50; Thermo Fisher Scientific Inc., USA) and subsequently with an Alexa 555-conjugated secondary antibody.

## Results

### A Reliable Method for AC Injection in Rodent

A correctly positioned air bubble minimized reflux after AC injection of AAV contained within a low viscosity storage buffer (BSS +Tween 20) (**Fig 1**). The air bubble was absorbed within 24 hrs. and did not harm the ocular tissues. No signs of inflammation were observed after injection. Although care was taken to avoid any irritation of ocular tissues, we assume that the cornea of 1 mouse and 1 rat eye (in total 18 rats and 18 mice were injected; 3 animals of each species served as untreated control) may have been disturbed during the injection procedure, thus leading to inadvertent intrastromal delivery of AAV vectors and transduction of corneal stromal keratinocytes.

### scAAV2-based Vectors Efficiently Transduce Tissues of the Anterior Segment

**Fig 2:**
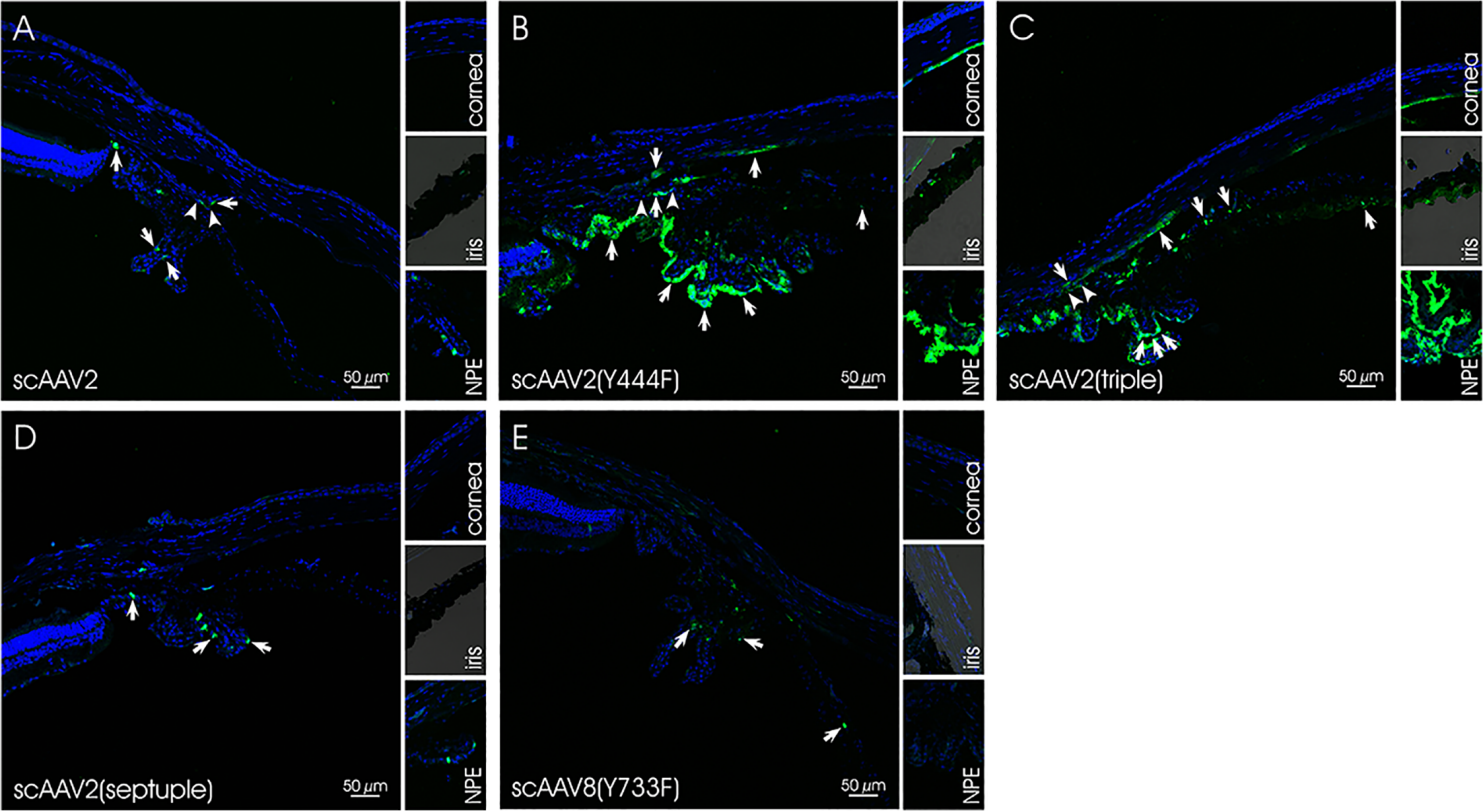
GFP expression in scAAV-injected mouse eyes. In A-D, representative sections of the region around the chamber angle are shown. The small inserts illustrate the cornea, the iris and the NPE. Nuclei are stained with DAPI. Arrows indicate GFP signals in the cornea, the iris, the chamber angle, the ciliary body and/or the NPE. Arrowheads mark the region of the TM.

**Fig 3:**
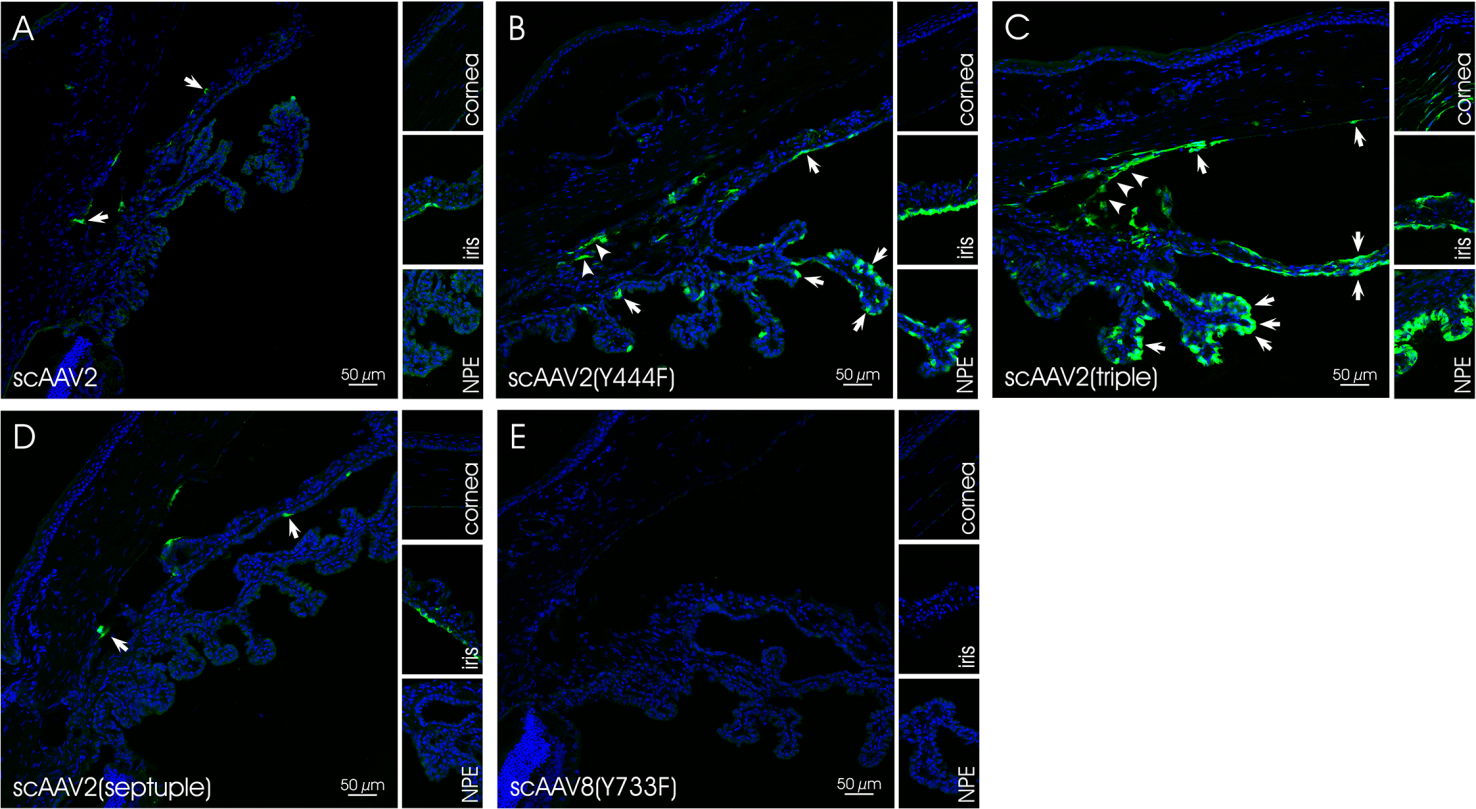
GFP expression in scAAV-injected rat eyes. In A-D, representative sections of the region around the chamber angle are shown. The small inserts illustrate the cornea, the iris and the NPE. Nuclei are stained with DAPI. Arrows indicate GFP signals in the cornea, the iris, the chamber angle and/or the NPE. Arrowheads mark the region of the TM.

**Fig 4:**
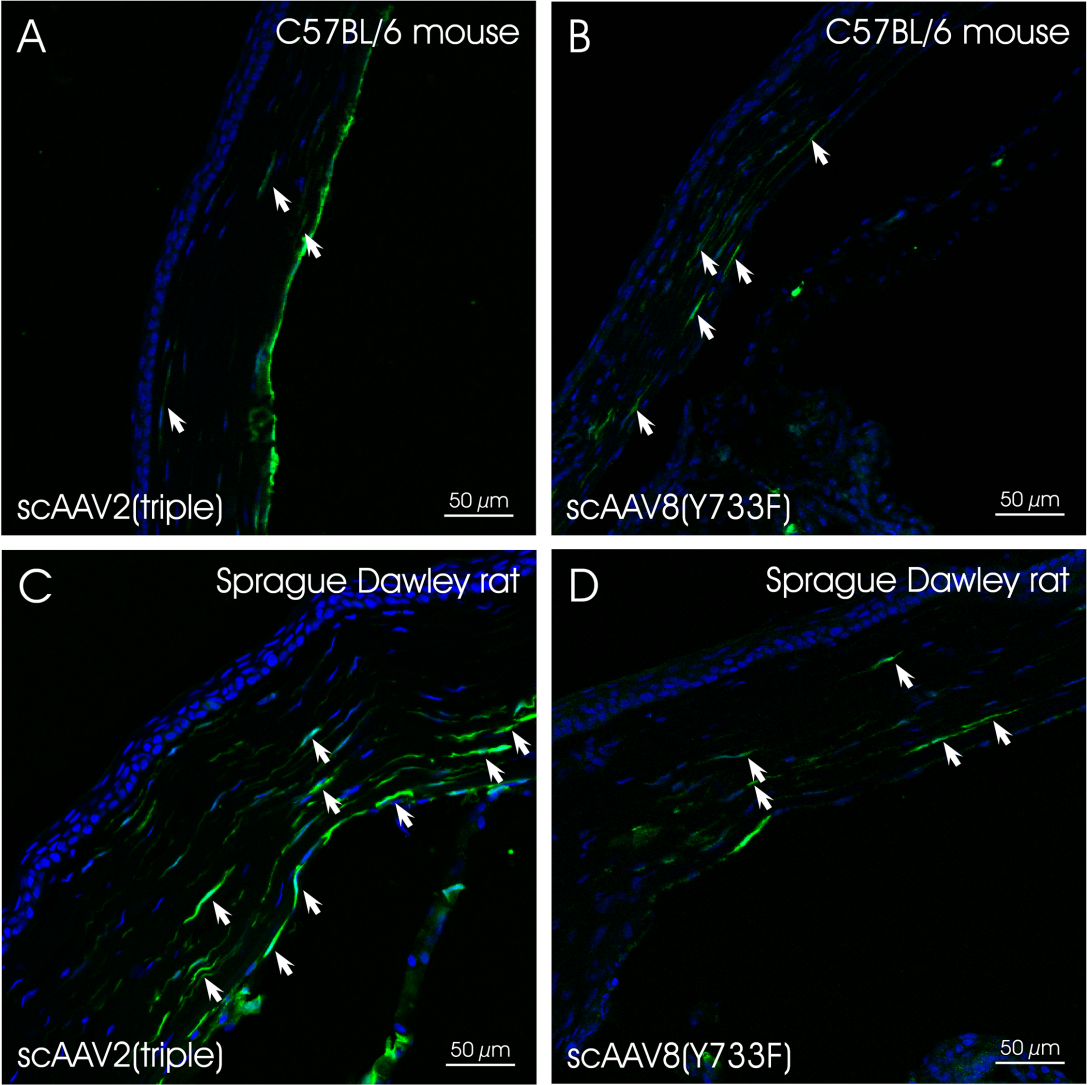
Corneas of scAAV2(triple)- and scAAV8(Y733F)- injected animals. GFP expression was detected in (A) scAAV(triple) and (B) scAAV8(Y733F)- injected mouse as well as in (C) scAAV2(triple)- and (D) scAAV8(Y733F)- injected rat corneas. Arrows indicate GFP- positive keratinocytes.

**Fig 5:**
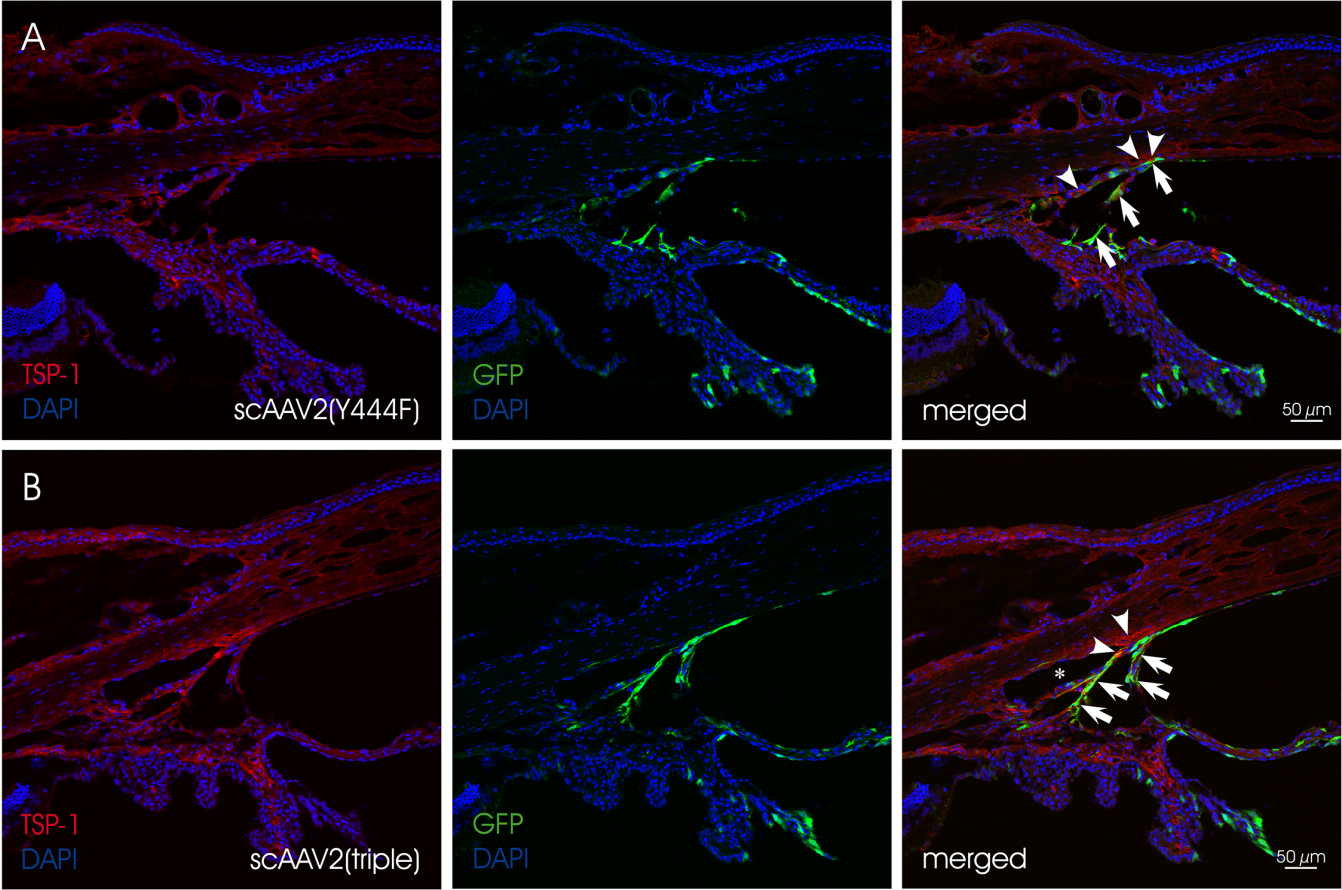
Co-staining of GFP (green, arrows) and TSP-1 (red, arrowheads) in chamber angle of injected rat eyes. (A) scAAV2(Y444F) and (B) scAAV2(triple) mediate efficient transgene expression in the region of the trabecular meshwork. Nuclei are stained with DAPI. Asterisk indicates Schlemm‘s canal.

At four weeks post injection, eyes injected with scAAV2-based vectors exhibited GFP expression in the corneal endothelium, the ciliary non-pigmented epithelium (NPE), the iris and the chamber angle including the TM (**Fig 2**and **Fig 3**). Localization and semiquantitative evaluation of the GFP-signal in injected eyes are summarized in Table 3. For the semiquantitation, we used a grading system based on the presence of GFP-positive cells/structures in the anterior segment and the distribution of GFP-signal. Similar grading systems have previously been used to evaluate viral vector-mediated transduction of ocular tissues, including AAV [76, 77]. Four scores were defined: (1) no immunopositivity (-), (2) isolated positive cells/structures (-/+), (3) isolated to mosaic-like immunopositivity of cells/structures (+) and (4) mosaic-like to almost homogenous immunopositivtiy (++). The overall score was based on analysis of eight, 10 micron sections per eye by three independent examiners. Representative sections were chosen for illustration. The data show that scAAV2(Y444F) and scAAV2(triple) were highly efficient in transducing tissues of the anterior segment, whereas scAAV2 and scAAV2(septuple) showed only low efficiency. scAAV2(Y444F) and scAAV2(triple) had titers of ~9 x 10^11^ and ~3 x 10^12^, respectively (Tables 1 and 3). It is possible that the slightly higher transduction efficiency observed with the triple vs. the single mutant resulted from the former being injected at a higher dose. In one out of five scAAV2(triple)- (**Fig 4A** and **Fig 4B**) and scAAV8(Y733F)- (**Fig 4C** and **Fig 4D**) injected eyes, GFP expression was detected in corneal stromal keratinocytes, a result likely owed to inadvertent intrastromal delivery. scAAV2(triple)-mediated GFP expression was more robust than that of scAAV8(Y733F). However, in general, GFP expression was absent from rodent eyes injected with scAAV8(Y733F). Depending on the scAAV variant used, the GFP-expression pattern ranged from isolated cells/structures to mosaic-like and homogenous (**Fig 2**and **Fig 3**). GFP was absent from both mock- injected (vehicle only) and uninjected eyes. Co-staining with TSP-1 in rat indicates that scAAV2(Y444F) and scAAV2(triple) transduced the TM structures (**Fig 5**). Both scAAV2(Y444F) and scAAV2(triple) transduced NPE of mice and rats very effectively. In contrast, scAAV2 and scAAV2(septuple) transduced only mouse NPE indicating that species differences may exist. No GFP-expression was present in the NPE of scAAV8(Y733F)- injected animals. Transduction of iris also appeared partly dependent on the species (**Table 3**). Corneal endothelium of mice and rats was effectively transduced by scAAV2(triple), whereas scAAV2(Y444F) only transduced corneal endothelium in mice efficiently.

**Table 3:** Localization of the GFP signal in the anterior section of eyes injected with similar titers of scAAVs.

	Vector	C57BL/6 Mice (Injection Vol 1 μL)	Sprague Dawley Rats (Injection Vol 2 μL)
Group 1	scAAV2 WT~2x10^12^	cornea—NPE + chamber angle + iris -	cornea—NPE—chamber angle + iris +
Group 2	scAAV2(Y444F) ~9x10^11^	cornea + NPE ++ chamber angle ++ iris+	cornea—NPE ++ chamber angle ++ iris +
Group 3	scAAV2(triple) ~3x10^12^	cornea ++*NPE ++ chamber angle ++ iris +	cornea ++*NPE ++ chamber angle ++ iris +
Group 4	scAAV(septuple) ~2x10^12^	cornea—NPE-/+ chamber angle—iris -	cornea—NPE—chamber angle-/+ iris-/+
Group 5	scAAV8(Y733F) ~2x10^12^	cornea -**NPE—chamber angle—iris-/+	cornea -**NPE—chamber angle—iris -

The transduction efficiency (grading:-,-/+, +, ++) is based on the intensity and distribution of the GFP signal in tissues of the anterior section. Unless otherwise noted (see asterisks) the term cornea represents GFP-expression in the corneal endothelium.

* One out of five showed GFP-positivity in the corneal stroma in addition to the corneal endothelium.

** One out of five showed GFP-positivity in the corneal stroma.

## Discussion

In this study, we established the transduction profiles of enhanced, capsid- mutated AAV vectors carrying self-complementary genomes following AC injection in mouse and rat. All previous reports of AAV-mediated transduction of AC tissues such as TM or corneal stroma have focused on unmodified, first generation serotypes AAV1-AAV9 [49, 51, 54–58]. However, the AAV vector toolkit is rapidly expanding and by combining knowledge of AAV capsid sequences with the structures available for these serotypes [78–83] (and unpublished data), it is now possible to delineate determinants of vector function (both intracellular and extracellular) and to rationally design vectors with desired biological properties [12, 84, 85]. For example, the three dimensional structure of AAV2 was used to identify capsid surface tyrosine, threonine, and serine residues, reported to promote ubiquitination and subsequent proteosomal degradation [67, 86]. Substitution of these residues significantly increased transduction efficiency and kinetics relative to unmodified virus in various tissues [65, 67, 87–89]. This approach was recently extended to the mutagenesis of lysine residues, which are directly ubiquitinated [86]. We have shown that, when subretinally delivered, Y-F mutants can restore function and preserve structure in multiple mouse models of retinal disease including the GC1KO and GCDKO mouse models of Leber congenital amaurosis-1 and the otherwise refractory *rd10* mouse model of autosomal recessive retinitis pigmentosa [90–92]. We chose to test similar AAV capsid variants for their ability to effectively transduce tissues in the anterior chamber such as TM and cornea. Because previous reports suggest that self-complimentary genomes are a requirement for TM transduction, we focused here only on scAAV vectors. Their ability to bypass rate-limiting second-strand DNA synthesis to obtain the transcriptionally active AAV genome results in earlier onset of transgene expression and thus a more rapid readout [93].

In previous studies, unmodified AAV2 and AAV8 vectors proved capable of targeting TM (intracameral injection) and cornea (intrastromal injection), respectively [49, 51, 54, 57]. This is likely owed to their respective receptor biology and the glycan footprint in target tissues. AAV2 binds HSPG, a proteoglycan abundant in TM and present in the cornea [59, 60]. AAV2’s co-receptor, αVβ5 integrin is also found in the extracellular matrix of TM [61]. Conversely, AAV8 does not bind HSPG [63]. It is not surprising, therefore, that scAAV2(Y444F) and scAAV2(triple) vectors mediated relatively high levels of GFP expression in mouse and rat TM. Localization of AAV-mediated GFP signal in the TM was demonstrated by double-labeling with TSP-1 in rats. A previous study in rat [51] showed that AC-injected scAAV2 containing GFP driven by the human enhanced cytomegalovirus (CMV) promoter resulted in efficient transgene expression only after a period of 2.5 months. In contrast, our results show that scAAV2(Y444F)- and scAAV2(triple)- mediated GFP expression is robust by 4 weeks post-injection. As we did not evaluate transduction beyond 4 weeks we cannot determine conclusively whether scAAV2(Y-F) mutants simply lead to faster onset of expression. However, we note in other ocular tissues (i.e. retinal ganglion cells, photoreceptors and retinal pigment epithelium), AAV2(Y-F) vectors consistently promote higher levels of transgene expression, for which early onset is the lead indicator [12, 13, 89, 94]. In our study we utilized the AAV8 capsid mutant (Y733F). While a direct comparison to unmodified AAV8 was not performed, existing data suggests that addition of the Y733F mutation does not result in changes of tropism [13]. We would therefor expect that un-modified scAAV8 vectors would lead to the same transduction pattern observed in this study.

An open question is whether efficient TM transduction relies on the use of self-complimentary genomes. Previously, Borras and colleagues found that co-infection of scAAV and recombinant Adenovirus with E1 and E3 deleted (rAdΔE1/E3) led to efficient transduction of TM [54]. They determined that single stranded AAV infection reduced expression of genes associated with DNA synthesis in the TM, which was reversed by the addition of rAdΔE1/E3. Use of scAAV vectors would overcome this rate limiting step and were hence tested. Interestingly, it has been shown that co-infection of AAV with empty Adenovirus capsid results in increased nuclear translocation of AAV [95]. Mechanistically, this is the same rationale for enhancement by the AAV(Y-F) variants [96]. It remains to be seen if these enhanced AAV2 capsid variants can overcome the need for utilization of a self-complementary gene cassette. This is of interest, as the canine model of spontaneous primary open angle glaucoma (POAG beagle), is associated with a mutation in the metalloproteinase ADAMTS10, which has a cDNA of 3312 bases, too large to be accommodated by scAAV vectors [97].

In addition to defining the transduction profile of scAAV2- and scAAV8- based capsid variants after AC injection, we also describe in detail a reliable AC injection technique for injecting low viscosity material in in both rat and mouse. This technique minimizes reflux, a common hurdle faced when delivering vector to this shallow chamber, and is reproducible, as evidenced by the repeatability of transduction by the efficient scAAV2 variants. Coupled with the diversity of genetically modified mouse strains, this approach will aid in elucidating biological processes of the anterior segment. Additionally, it impacts the development of gene-based therapies for the treatment of glaucoma and corneal disease/injury.
